# Effect of Oral Insulin on Early Combined Glucose and C-Peptide Endpoints in Individuals at High-Risk for Type 1 Diabetes

**DOI:** 10.1155/2024/8343868

**Published:** 2024-10-03

**Authors:** Taylor M. Triolo, Laura M. Jacobsen, David Cuthbertson, Emily K. Sims, Heba M. Ismail, Maria J. Redondo, Markus Lundgren, Linda A. DiMeglio, Peter A. Gottlieb, Mark A. Atkinson, Jeffrey P. Krischer, Desmond A. Schatz, Jay M. Sosenko

**Affiliations:** ^1^ The Barbara Davis Center for Diabetes University of Colorado Anschutz Medical Campus, Aurora, Colorado, USA; ^2^ Division of Pediatric Endocrinology University of Florida, Gainesville, Florida, USA; ^3^ Health Informatics Institute University of South Florida, Tampa, Florida, USA; ^4^ Department of Pediatrics Division of Pediatric Endocrinology and Diabetology Indiana University, Indianapolis, Indiana, USA; ^5^ Section of Diabetes and Endocrinology Texas Children's Hospital Baylor College of Medicine, Houston, Texas, USA; ^6^ Unit for Pediatric Endocrinology Department of Clinical Sciences Malmö Lund University, Malmö, Sweden; ^7^ Department of Pathology Immunology and Laboratory Medicine University of Florida, Gainesville, Florida, USA; ^8^ Division of Endocrinology University of Miami, Miami, Florida, USA

**Keywords:** C-peptide, oral insulin, prevention, type 1 diabetes

## Abstract

**Background:** The TrialNet Oral Insulin (OI) prevention trial showed no overall treatment effect, using the diagnosis of type 1 diabetes as an endpoint. A significant delay in onset was only found in a high-risk stratum (termed secondary stratum 1) of participants with low first-phase insulin release (FPIR).

**Methods:** Since trials with an endpoint of type 1 diabetes take years to complete, in this post hoc analysis, we assessed whether a novel combination of glucose and C-peptide markers could identify a therapeutic benefit after 1 year of follow-up (trial participants followed for a median 2.7 years).

**Results:** Participants were relatives with multiple islet autoantibodies and low FPIR (*n* = 40). Glucose rose, and C-peptide declined in the placebo group, whereas glucose rose minimally, and C-peptide increased in the OI group. When glucose and C-peptide were plotted on two-dimensional grids using 30–120-min oral glucose tolerance test (OGTT) time points, changes in ratios of their central points (centroid ratio) differed between groups (*p*=0.037 adjusted for age, BMI, and baseline C-peptide and glucose).

**Conclusions:** These findings support a favorable early effect of OI on combined glucose and C-peptide endpoints in high-risk individuals, indicating metabolic benefit. With further study, these measures may allow for shorter trials compared to the standard endpoint of type 1 diabetes diagnosis.

## 1. Introduction

Even among high-risk multiple autoantibody-positive individuals, type 1 diabetes prevention trials can take years to complete due to the time for type 1 diabetes onset to occur. TrialNet conducted a large, multicenter, randomized, placebo-controlled trial to assess whether oral insulin (OI) could prevent or delay diabetes onset. This study was designed to encompass multiple strata. The primary stratum (PS) included relatives with multiple autoantibodies and normal first-phase insulin response (FPIR) on intravenous glucose tolerance testing (IVGTT). Within the PS, individuals who received OI had a similar rate of type 1 diabetes compared to those who received placebo (i.e., no overall therapeutic benefit) [1]. In addition, there was a planned exploratory subgroup, named secondary stratum 1 (SS1), which included participants with a similar autoantibody profile but low FPIR. In SS1, participants in the OI group had a lower rate of diabetes than those in the placebo group [1].

Combination markers, such as Index60 [2–4] or area under the curve (AUC) C-peptide/AUC glucose (AUC ratio), can provide more specificity regarding beta-cell function and prediction of type 1 diabetes progression than either glucose or C-peptide alone. Also, in post hoc analyses of the high-risk participants in the Diabetes Prevention Trial-Type 1 (DPT-1) OI trial and TrialNet OI trial PS with AUC ratio after 1 year of follow-up as the endpoint, the OI groups in both trials appeared to have delayed metabolic progression [5]. Since combination markers appear to better characterize the progression to type 1 diabetes [6], we sought to assess those and other measures as indicators of a therapeutic benefit earlier, after 1 year, in the TrialNet OI SS1 prevention trial [7].

## 2. Research Design and Methods

The multicenter Type 1 Diabetes TrialNet OI study (NCT00419562) included multiple autoantibody-positive relatives of individuals with type 1 diabetes who were treated with 7.5 mg/day of OI or placebo for a median follow-up of 2.7 years and monitored for diabetes development. At-risk relatives had either (1) insulin autoantibodies (mIAA) and islet cell autoantibodies or (2) mIAA with insulinoma-associated 2 autoantibodies (IA2A) and glutamic acid decarboxylase autoantibodies. Participants in SS1 (*n* = 55) had normal glucose tolerance on oral glucose tolerance testing (OGTT) but low FPIR on IVGTT, where the sum of the 1- and 3-min insulin values was <60 μU/mL for parents of probands and relatives <8 years old or <100 μU/mL if relatives (other than parents) were ≥8 years old as previously described [1].

OGTTs were performed every 6 months until the development of type 1 diabetes, based on the American Diabetes Association criteria, or conclusion of the trial. Glucose and C-peptide measurements were obtained at 0, 30, 60, 90, and 120 min after ingestion of a 1.75 g/kg carbohydrate drink (maximum 75 g). Participants were typed for HLA class II DRB1, DQA1, and DQB1 alleles using DNA-based typing with oligonucleotide probes, as previously described [8, 9]. Subjects were stratified by the presence of the high-risk HLA genotype DR3 DQA1 ^*∗*^05:01 DQB1 ^*∗*^02:01/DR4 DQA1 ^*∗*^03:01 DQB1 ^*∗*^03:02 (abbreviated here DR3/4 ^*∗*^0302).

Glucose and C-peptide response curves (GCRCs) from two-dimensional plots of OGTT C-peptide and glucose mean values at 30, 60, 90, and 120-min were used [10] to visually depict treatment differences between OI and placebo. Combination glucose and C-peptide endpoints studied included (1) AUC ratio, (2) Index60, and (3) the newly developed centroid ratio. The AUC ratio includes all time points from the 120-min OGTT and a decrease in the measure denotes worsening beta-cell function. Index60, developed from DPT-1 data using a proportional hazards regression model, is less than zero in a metabolically healthy state (0.3695 × [log fasting C-peptide] + 0.0165 × [60-min glucose] − 0.3644 × [60-min C-peptide]) [2]. A centroid is the central point of the GCRC shape, the latter constituted by 30–120-min OGTT time points and was calculated as reported in Sims et al. [11]. The C-peptide and glucose coordinates that define the centroid location are used to form the centroid ratio (C-peptide coordinate/glucose coordinate); the centroid ratio can be compared between groups and over time. A decrease in the measure is indicative of a worsening metabolic state. Other noncombination measures were also assessed: AUC C-peptide, AUC glucose, 30–0 min C-peptide, and peak C-peptide (highest value from the 120-min OGTT).

To provide comparisons with previous studies, the mean AUC glucose and mean AUC C-peptide values at baseline and 1 year were plotted. A vector connecting these points allowed for a scaled angle that is determined from the right horizontal line/*x*-axis (0°). This same plot was utilized in the prior report [5] that compared placebo and OI groups in both the TrialNet PS and DPT-1 OI trial cohorts that had an elevated metabolic risk (DPT-1 risk score [DPTRS] ≥ 6.75), and the differences in vector angles were determined.

The change in each metabolic measure from baseline to 1 year was compared between the placebo and OI arms. Participants were excluded if they did not have baseline or 12-month data or if they developed type 1 diabetes prior to or at 12 months (*n* = 15). Of these, seven were removed due to missing data at baseline or 12 months (three placebo; four treated) and eight due to development of diabetes at or before 12 months (five placebo; three treated). Adjustments using ANOVA were conducted by including baseline age, BMI Z-score, C-peptide, and glucose in the regression model. ANOVA was used to compare the continuous measures (age, BMI Z-score, and metabolic measures), while the chi-square statistic was used for comparing categorical variables (sex and race/ethnicity). *p*-Values less than 0.05 were considered significant. All statistical analyses were performed using SAS version 9.4 (Cary, NC).

## 3. Results

Of the 40 subjects included in this analysis of TrialNet OI SS1, 19 were in the placebo arm and 21 in the OI arm. Seventy-three percent of participants (29/40) went on to develop type 1 diabetes after the initial 12 months (79% in the placebo group and 67% in the OI group). Participants were followed for 3.5 ± 1.9 years (mean ± SD). Baseline characteristics are shown in Table 1. There were no significant differences in age, sex, and race/ethnicity between those who received placebo and those treated with OI. BMI Z-scores were 0.10 ± 0.65 in the placebo treated subjects and –0.45 ± 0.92 in the OI treated subjects (*p*=0.048). There was no difference in the presence of the high-risk HLA haplotypes. At baseline, the combination and noncombination metabolic markers were not significantly different between placebo and OI.

**Figure 1:**
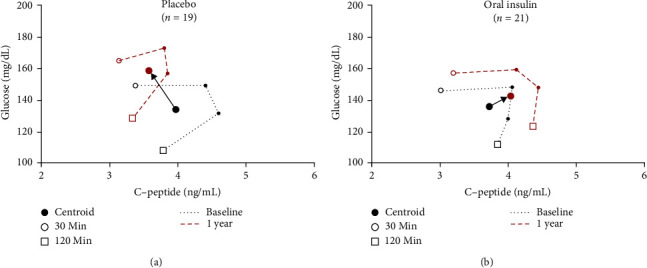
Glucose C-peptide response curves (GCRCs) from mean C-peptide and glucose values at 30, 60, 90, and 120-min time points of an OGTT for participants in the TrialNet Oral Insulin SS1 (A) placebo and (B) oral insulin groups. Baseline values (black dotted line) and 1-year values (red dashed line) are depicted with centroids plotted for each curve and a vector connecting baseline to 1 year.

We analyzed mean glucose and C-peptide values at 30, 60, 90, and 120-min time points from OGTTs performed at baseline and 1 year of follow-up, using GCRCs on two-dimensional grids. The 1-year time from baseline was chosen since an OI effect was evident at that time point in the prior study of OI [1].  Figure 1A shows the change from baseline to 1 year in those who received placebo. Over the year, the mean glucose increased, and the mean C-peptide decreased at all time points. In contrast, among those treated with OI (Figure 1B), the mean glucose increased marginally, while the mean C-peptide increased at all time points. This treatment effect is visually demonstrated by the difference between the placebo and OI groups in the directionality of the vectors from the centroid at baseline to the centroid at 1 year.

We examined the change in mean values for combination and noncombination glucose and C-peptide markers from baseline to 1 year of follow-up (Table 2). Unlike changes in AUC ratio and Index60, the centroid ratio demonstrated significant difference from baseline to 1 year. Those given placebo had a change in centroid ratio of –0.005 ± 0.01 compared to those treated with OI 0.001 ± 0.01 (*p*=0.018 unadjusted, *p*=0.037 adjusted). Measures in which C-peptide and glucose were not combined (AUC C-peptide, AUC glucose, 30–0 min C-peptide, or peak C-peptide) did not differ significantly between treatment arms over 1 year. Hazard ratios (and 95% confidence intervals) were calculated using univariable Cox models for the change in each metabolic measure from baseline to 12 months and the eventual risk of diabetes development (*n* = 29 with complete data). Only Index60 (HR 2.049 [1.254, 3.350], *p*=0.004) and AUC glucose (HR 1.013 [1.003, 1.024], *p*=0.014) were associated with progression to type 1 diabetes.

**Figure 2:**
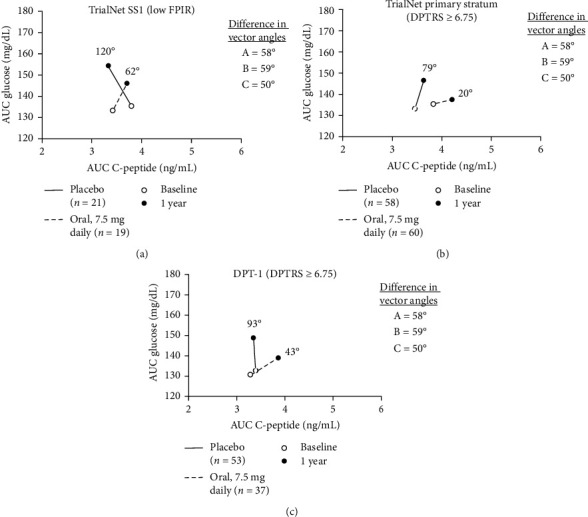
One-year directional change in glucose and C-peptide area under the curve (AUC) in oral insulin prevention trials: (A) TrialNet SS1 (low FPIR), (B) TrialNet Primary Stratum (DPTRS ≥ 6.75), and (C) DPT-1 Oral Insulin (DPTRS ≥ 6.75). A vector connects mean baseline AUC values (open circle) and 1-year AUC values (closed circle) for placebo (solid line) and oral insulin (dashed line) groups. Parts of this figure were published previously in Sosenko et al. [5] *Slowed Metabolic Decline After 1 Year of Oral Insulin Treatment Among Individuals at High Risk for Type 1 Diabetes in the Diabetes Prevention Trial-Type 1 (DPT-1) and TrialNet Oral Insulin Prevention Trials*. Diabetes. 2020 Aug;69(8):1827−1832. Copyright 2020 by the American Diabetes Association.

The difference between the placebo and OI treatment group vectors in SS1was compared with the vector differences previously observed in the analyses of TrialNet PS and DPT-1 (5). Since mean AUC glucose and mean AUC C-peptide were the bidirectional axes used for plotting vectors in that study, we utilized the same axes to plot the vectors in SS1 (Figure 2). The angle of the difference between the placebo and OI vectors from baseline to 1 year in SS1 (58°) was similar to the angle of the difference between the placebo and OI vectors from baseline to 1 year in analyses of those at high risk in the PS (59°) and DPT-1 (50°) trials.

## 4. Discussion

A difference at 1 year between placebo and OI groups in the TrialNet OI SS1 was detected quantitatively by the change in the centroid ratio, a newly developed combination glucose and C-peptide measure. In that year, the vectors on the two-dimensional grid showed the GCRC of the placebo group moved in a pathological direction, upward and leftward (i.e., increasing glucose and decreasing C-peptide). In contrast, the GCRC of the OI group moved only slightly upward and rightward. These findings illustrate the importance, both quantitatively and qualitatively, of assessing glucose and C-peptide in the context of each other.

Previously, prevention studies have focused on the clinical outcome of the development of type 1 diabetes in high-risk relatives [12]. The centroid ratio, a combination C-peptide and glucose marker, differed between treatment arms earlier than the clinical outcome of type 1 diabetes—at 1 year versus the average time to diabetes in SS1 which was 2.65 years. Thus, the centroid ratio has potential utility, in research, as an early signal of efficacy in prevention trials which could save time and money.

OI trials have not met primary endpoints to delay or prevent type 1 diabetes in those at risk [1, 13]. However, subgroups within these trials have shown some benefit [13–15]. Subjects with high IAA titers in DPT-1 and those with loss of FPIR in TrialNet (the cohort study herein) in post hoc analyses are suggested to have delayed progression to type 1 diabetes [1]. Our study is consistent with a prior analysis, which showed evidence (using AUC ratio) suggesting that individuals at high risk (i.e., DPTRS ≥6.75) benefitted from OI therapy [5]. Since SS1 was preplanned and showed delayed progression to diabetes in the OI group, the metabolic findings at 1 year can be viewed as corroborative. This suggests that measurements of early metabolic endpoints could be of value if specified in protocols for prevention trials.

The two-dimensional grid of mean AUC glucose and mean AUC C-peptide provided the basis for this unique, qualitative means of interpreting prevention trial outcomes. Visual evidence for the similarities in the findings from analyses of TrialNet SS1 and the two other OI trials [5] is apparent. The difference in vector angles for change between the OI and placebo groups was similar for the three studies (DPT-1, 50°; TrialNet PS, 59°; and SS1, 58°).

This study was limited by small participant numbers available for analysis. Multiple test corrections were not performed due to the exploratory nature of this work, and confirmatory studies are needed. As indicated above, the metabolic endpoint findings are consistent with the high risk (DPTRS ≥6.75) DPT-1 and TrialNet PS participants. Similarly, combined glucose and C-peptide endpoints provided evidence of a teplizumab effect at 3 months [11]. Thus, the analyses of combination metabolic endpoints in SS1 would appear to add to the growing evidence that combination metabolic endpoints are useful for showing early efficacy of preventive treatments in therapies that have thus far been unsuccessful. Future prospective studies could be stratified by these metabolic criteria to further evaluate the utility of these measures.

## 5. Conclusions

Overall, in the large trials, OI did not delay or prevent type 1 diabetes. However, post hoc analyses of those trials and of SS1 suggest that individuals at high-risk (e.g., low FPIR and elevated DPTRS) can derive the benefit of delayed metabolic progression from OI. Our findings support the further study of such combination glucose and C-peptide measures as short-term endpoints in prevention trials. Continued advancement of OI for clinical application should be considered, either alone or in combination with other immunomodulatory agents.

## Figures and Tables

**Table 1:** Baseline characteristics of oral insulin and placebo-treated participants.

Mean ± SD unless otherwise stated	Placebo (*n* = 19)	Oral insulin (*n* = 21)	*p*-Value
Age, years	9.74 ± 5.48	10.14 ± 4.26	0.800
% Male/female	79/21	76/24	0.835
Race/ethnicity, *N* (%)			0.991
NHW	16 (84.2%)	18 (85.7%)	
Hispanic	1 (5.3%)	1 (4.8%)	
Other	2 (10.5%)	2 (9.5%)	
HLA			0.590
DR3/4 ^*∗*^0302	3 (15.8%)	4 (19.0%)	
DR3 only	3 (15.8%)	1 (4.8%)	
DR4 ^*∗*^0302 only	9 (47.4%)	13 (61.9%)	
DRX/X (neither DR3 nor DR4 ^*∗*^0302)	4 (21.0%)	3 (14.3%)	
BMI Z-score	0.10 ± 0.65	−0.45 ± 0.92	**0.048**
Combination markers			
AUC ratio	2.84 ± 1.15	2.66 ± 0.92	0.583
Index60	0.81 ± 0.69	0.94 ± 0.75	0.599
Centroid ratio	0.030 ± 0.012	0.029 ± 0.011	0.698
Noncombination markers			
AUC C-peptide (ng/mL)	3.79 ± 1.41	3.42 ± 0.93	0.324
AUC glucose (mg/dL)	134.96 ± 14.25	132.89 ± 20.37	0.715
30–0 min C-peptide (ng/mL)	2.38 ± 1.10	2.00 ± 1.00	0.264
Peak C-peptide (ng/mL)	5.00 ± 1.89	4.60 ± 1.26	0.441

*Note:* The bold values indicate *p* < 0.05.

**Table 2:** Change in mean values for three combination glucose and C-peptide markers and four noncombination glucose or C-peptide markers from baseline to 1 year.

Change in mean ± SD	Placebo (*n* = 19)	Oral insulin (*n* = 21)	*p*-Value (unadjusted)	*p*-Value (adjusted)
AUC ratio	−0.532 ± 0.86	−0.024 ± 0.68	**0.043**	0.108
Index60	0.615 ± 0.89	0.227 ± 0.76	0.145	0.108
Centroid ratio	−0.005 ± 0.01	0.001 ± 0.01	**0.018**	**0.037**
AUC C-peptide (ng/mL)	−0.469 ± 1.29	0.280 ± 1.33	0.079	0.360
AUC glucose (mg/dL)	19.34 ± 42.58	12.76 ± 32.29	0.583	0.440
30–0 min C-peptide (ng/mL)	−0.254 ± 1.19	0.016 ± 1.23	0.484	0.882
Peak C-peptide (ng/mL)	−0.487 ± 1.78	0.237 ± 1.82	0.212	0.451

*Note:* The bold values indicate *p* < 0.05.

## Data Availability

The data used to support the findings of this study are available from the corresponding author upon request.

## Nomenclature

OI: Oral insulin
FPIR: First-phase insulin release
OGTT: Oral glucose tolerance test
PS: Primary stratum
IVGTT: Intravenous glucose tolerance testing
SS1: Secondary stratum 1
AUC: Area under the curve
AUC ratio: C-peptide/AUC glucose
DPT-1: Diabetes Prevention Trial-Type 1
GCRCs: Glucose and C-peptide response curves.

## References

[1] Study G., Krischer J. P., Schatz D. A., Bundy B., Skyler J. S., Greenbaum C. J. (2017). Writing Committee for the Type 1 Diabetes TrialNet Oral Insulin, Effect of Oral Insulin on Prevention of Diabetes in Relatives of Patients With Type 1 Diabetes: A Randomized Clinical Trial. *JAMA*.

[2] Sosenko J. M., Skyler J. S., DiMeglio L. A. (2015). A New Approach for Diagnosing Type 1 Diabetes in Autoantibody-Positive Individuals Based on Prediction and Natural History. *Diabetes Care*.

[3] Nathan B. M., Boulware D., Geyer S. (2017). Dysglycemia and Index60 as Prediagnostic End Points for Type 1 Diabetes Prevention Trials. *Diabetes Care*.

[4] Redondo M. J., Nathan B. M., Jacobsen L. M. (2021). Index60 as an Additional Diagnostic Criterion for Type 1 Diabetes. *Diabetologia*.

[5] Sosenko J. M., Skyler J. S., Herold K. C. (2020). Slowed Metabolic Decline After 1 Year of Oral Insulin Treatment Among Individuals at High Risk for Type 1 Diabetes in the Diabetes Prevention Trial-Type 1 (DPT-1) and TrialNet Oral Insulin Prevention Trials. *Diabetes*.

[6] Jacobsen L. M., Bocchino L., Evans-Molina C. (2020). The Risk of Progression to Type 1 Diabetes Is Highly Variable in Individuals With Multiple Autoantibodies Following Screening. *Diabetologia*.

[7] Jacobsen L., Cuthbertson D., Sims E. (2021). 589-P: Effect of Oral Insulin (OI) on Combined Glucose and C-Peptide Endpoints in Individuals at High-Risk for Type 1 Diabetes (T1D). *Diabetes*.

[8] Rewers A., Babu S., Wang T. B. (2003). Ethnic Differences in the Associations Between the HLA-DRB1 ^*∗*^04 Subtypes and Type 1 Diabetes. *Annals of the New York Academy of Sciences*.

[9] Mychaleckyj J. C., Noble J. A., Moonsamy P. V. (2010). HLA Genotyping in the International Type 1 Diabetes Genetics Consortium. *Clinical Trials*.

[10] Ismail H. M., Cleves M. A., Xu P. (2020). The Pathological Evolution of Glucose Response Curves During the Progression to Type 1 Diabetes in the TrialNet Pathway to Prevention Study. *Diabetes Care*.

[11] Sims E. K., Cuthbertson D., Herold K. C., Sosenko J. M. (2021). The Deterrence of Rapid Metabolic Decline Within 3 Months After Teplizumab Treatment in Individuals at High Risk for Type 1 Diabetes. *Diabetes*.

[12] Primavera M., Giannini C., Chiarelli F. (2020). Prediction and Prevention of Type 1 Diabetes. *Frontiers in Endocrinology*.

[13] Skyler J. S., Krischer J. P., Wolfsdorf J. (2005). Effects of Oral Insulin in Relatives of Patients with Type 1 Diabetes: The Diabetes Prevention Trial—Type 1. *Diabetes Care*.

[14] Bonifacio E., Ziegler A. G., Klingensmith G. (2015). Effects of High-Dose Oral Insulin on Immune Responses in Children at High Risk for Type 1 Diabetes: The Pre-POINT Randomized Clinical Trial. *JAMA*.

[15] Vehik K., Cuthbertson D., Ruhlig H., Schatz D. A., Peakman M., Krischer J. P. (2011). Long-Term Outcome of Individuals Treated with Oral Insulin: Diabetes Prevention Trial-Type 1 (DPT-1) Oral Insulin Trial. *Diabetes Care*.

